# Chromosome Y Haplogroup R Was Associated with the Risk of Premature Myocardial Infarction with ST-Elevation: Data from the CholeSTEMI Registry

**DOI:** 10.3390/jcm12144812

**Published:** 2023-07-21

**Authors:** Rebeca Lorca, Andrea Aparicio, María Salgado, Rut Álvarez-Velasco, Isaac Pascual, Juan Gomez, Daniel Vazquez-Coto, Claudia Garcia-Lago, Lucinda Velázquez-Cuervo, Elías Cuesta-Llavona, Pablo Avanzas, Eliecer Coto

**Affiliations:** 1 Área del Corazón, Hospital Universitario Central Asturias (HUCA), 33011 Oviedo, Spain; 2 Unidad de Cardiopatías Familiares, Área del Corazón y Departamento de Genética Molecular, Hospital Universitario Central Asturias, 33011 Oviedo, Spaineliecer.coto@sespa.es (E.C.); 3 Área de Fisiología, Departamento de Biología Funcional, Universidad de Oviedo, 33003 Oviedo, Spain; 4 Instituto de Investigación Sanitaria del Principado de Asturias (ISPA), 33011 Oviedo, Spain; 5 Redes de Investigación Cooperativa Orientadas a Resultados en Salud (RICORs), 28029 Madrid, Spain; 6 Departamento de Medicina, Universidad de Oviedo, 33003 Oviedo, Spain; 7 CIBER-Enfermedades Respiratorias, 28029 Madrid, Spain; 8 Genética Molecular, Hospital Universitario Central Asturias (HUCA), 33011 Oviedo, Spain; 9 Centro de Investigación Biomédica en Red de Enfermedades Cardiovasculares (CIBERCV), 28029 Madrid, Spain

**Keywords:** coronary artery disease, genetics, cardiovascular risk factors, premature cardiovascular disease, myocardial infarction

## Abstract

Cardiovascular disease (CVD) is the leading cause of death worldwide, with coronary artery disease (CAD) being one of its main manifestations. Both environmental and genetic factors are widely known to be related to CAD, such as smoking, diabetes mellitus, dyslipidemia, and a family history of CAD. However, there is still a lack of information about other risk factors, especially those related to genetic mutations. Sex represents a classic CAD risk factor, as men are more likely to suffer CAD, but there is lack of evidence with regard to sex-specific genetic factors. We evaluated the Y chromosome haplogroups in a cohort of young Spanish male patients who suffered from STEMI. In this cohort, haplogroup R was significantly more frequent in STEMI patients.

## 1. Introduction

Cardiovascular disease (CVD) remains the leading cause of morbidity and mortality worldwide [1,2], coronary artery disease (CAD) being a major concern. CAD is a complex disease with multiple risk factors, including genetics, lifestyle, and environmental factors [1,2]. Classic cardiovascular risk factors known to contribute to the development of CAD include smoking, high blood pressure, diabetes mellitus, high cholesterol levels or dyslipidemia (DL), a family history of CAD, obesity, a lack of physical activity, and stress [3]. Moreover, CAD has important genetic underpinnings that can be considered equivalent to environmental factors [4]. However, the role of genetic factors in CAD susceptibility, beyond familial hypercholesterolemia [5], is not yet well understood. In this regard, studying genetic susceptibility for premature CAD is of utmost importance.

Because CAD is more common among men, sex-specific factors might contribute to the risk of developing this disease. Although several loci have been identified to be associated with the genetic risk for CAD, the contribution of Y chromosome variants to CAD risk remains unclear. As a result, there has been increasing interest in the potential association between Y chromosome polymorphisms and CAD risk. Chromosome Y is male-specific and plays a determinant role in defining the biological characteristics of male sex. Chromosome Y is unique in that it is paternally inherited and passed down from father to son. Therefore, Y variants have been used to determine the patrilineal history and migration patterns of human populations [6,7]. In this regard, Y chromosome haplogroups are defined by specific sets of single-nucleotide polymorphisms (SNPs) that are passed down through the male lineage. The distribution of Y chromosome haplogroups varies significantly between populations due to differences in migration patterns, genetic drift, and natural selection, and reflects the timeline distance between human populations [6,8]. In this regard, haplogroup R (defined by SNP rs2032636, also known as M207) would have originated about 30,000 years ago in South-Asia and is present in more than half of Europeans. A subclade of R designated as R1b (SNP rs9786153 or M269) would have surged during the Upper Paleolithic (approx. 28,000 years ago) in the Caucasus region and is present in more than half of Spanish men, with higher frequencies in the Cantabrian cornice.

Some studies have suggested that Y chromosome haplogroups may also be associated with various health outcomes, including cancer or cardiovascular disease [9]. Some studies have reported an association between specific haplogroups and CAD risk [10,11]. However, other studies have failed to find such an association. In summary, the evidence linking specific haplogroups with CAD risk is still limited and controversial.

Many studies are limited by their sample sizes, differences in the definitions of CAD, presentation ages, ethnicities of the populations studied, and other potential confounding factors. Therefore, further studies are needed to elucidate the potential role of Y chromosome haplogroups in the development of CAD. Identifying genetic markers associated with CAD risk may help to develop more effective prevention and treatment strategies for this common and debilitating disease.

In this scenario, aiming to provide some insights into the genetic determinants of premature CAD in men, we aimed to evaluate the different Y chromosome haplogroups in a cohort of male patients presenting with premature myocardial infarction with ST-elevation (STEMI).

## 2. Materials and Methods

### 2.1. Study Population

Patients were recruited from the CholeSTEMI registry [5,12]. This study was approved by the local Ethical Committee (CEIMPA; registry number 2020/003) [5,12]. All participants who wished to participate in the actual investigational project had signed written consent to grant access to their genetic data for additional investigational purposes.

In this study, we review all consecutive patients referred to our center for emergency cardiac catheterization due to STEMI as reported elsewhere [5,12]. From the initial cohort of 157 male patients with premature STEMI, 35 were not studied due to lack of DNA. The main characteristics of the remaining 122 male patients with premature STEMI are shown in Table 1. All patients from this cohort were of European ancestry and from the region of Asturias (Northern Spain, total population of about 1 million). Only patients with type 1 myocardial infarction and confirmed atherothrombotic CAD by coronary angiogram, who agreed to participate in the CholeSTEMI registry, were included in this study. Patients with other kinds of myocardial infarction different to type 1 myocardial infarction (such as vasospasm, demand/supply mismatch due to hypoxemia, anemia or arrythmia, etc.), according to ESC guidelines [13,14], were excluded from analysis.

Premature CAD for men was considered if presented before the age of 55 [15,16]. Patients without sufficient stored DNA to perform the additional Y chromosome haplogroup study were excluded from this study.

Clinical data from this cohort was already reviewed: birth date, gender, age at STEMI, and classical cardiovascular risk factors: high blood pressure, tobacco consumption, diabetes mellitus, dyslipidemia, and premature CAD.

### 2.2. Control Cohort

We compare the Y chromosome haplogroup prevalence to that found in a representative control cohort of 200 individuals from the same region of the cohort of premature CAD presenting with STEMI. These male controls were aged <55 years and recruited as part of the RENASTUR project to determine the prevalence of cardiovascular risk factors in the region of Asturias [17]. For the objectives of our study, these controls were genotyped with the only purpose of determining the frequency of the main Y haplogroups in our population.

### 2.3. Genetic Testing

All the male patients (N = 122) and controls (N = 200) were genotyped for 8 single nucleotide polymorphisms in chromosome Y that defined the common European Y haplogroups (Table 1). The corresponding nucleotides were determined by real-time PCR with Taqman assays in an ABI7500 equipment and following the manufacturer instructions (Fisher Scientific). The allele frequencies among Europeans were obtained from the Ensembl database (www.ensembl.org).

### 2.4. Statistical Analysis

Statistical analysis was performed with STATA. Descriptive data for continuous variables are presented as mean ± SD and as frequencies or percentages for categorical variables. The Chi-square test was used to compare frequencies, whereas differences in continuous variables were evaluated with either Student’s *t*-test or Mann–Whitney U test. A *p* value below 0.05 was considered to be significant.

## 3. Results

The eight single nucleotide polymorphisms determined to define the Y haplogroups are summarized in Table 1. The frequency of the allele that defined the haplogroup among Europeans is indicated in Table 1. The haplogroup labelled as “other” corresponded to the remaining haplogroups that could not be assigned based on the allele combinations at the 8 SNPs.

**Figure 1:**
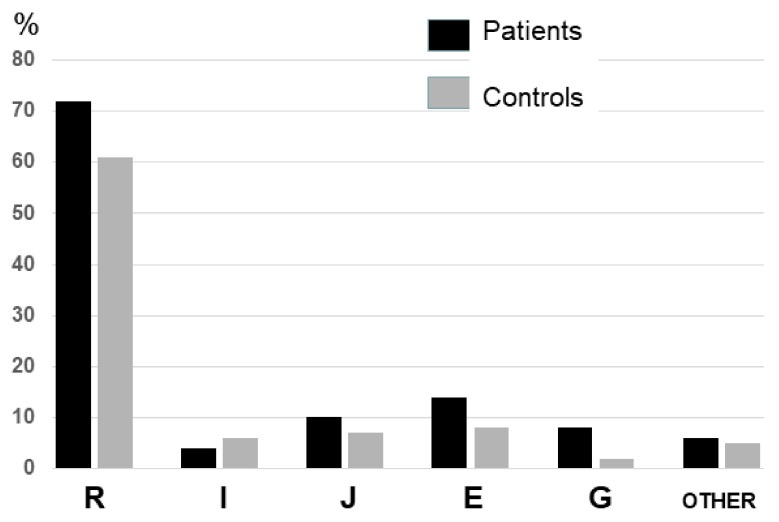
Prevalence of the different Y chromosome haplogroups in both control and patient’s cohorts.

Prevalence of the different Y chromosome haplogroups in both control and patient’s cohorts are shown in Figure 1. As expected, due to its geographical location in the Cantabrian cornice of Spain, haplogroup R was the most frequent in our population, followed by haplogroups E, H and G (Table 2). Due to it high frequency, we also evaluated R subhaplogroups, being most for them were R1 (SNP rs2032624 = M173) and R1B (Table 2).

However, only 2% of the controls and none of the STEMI patients were R1A. Moreover, when we compared the frequencies of R haplogroup between patients and controls, we found that cases had a significantly increased frequency (*p* = 0.04), with an OR = 1.65 (95% CI = 1.02–2.69). In particular, we found the frequency of Y chromosome haplogroup R1B in STEMI patients to be significantly higher than in the control cohort. Thus, in our population the common Y haplogroup R seems to be a risk factor for premature STEMI in men.

In contrast, in our region, the haplogroup I was rare, representing only 4% and 6% of the control and patient cohort, respectively (*p* = 0.47). Given its low frequency, subgroups of this haplogroup were not evaluated.

Haplogroups J, E and “other” showed a frequency that was non-significantly lower in the patients than in the controls, whereas haplogroup G was significantly less frequent (Table 2). For these rare haplogroups, we believe that evaluating the effect on STEMI risk is statistically underpowered due to the reduced sample size.

General clinical characteristics of the included 122 male patients with premature STEMI are shown in Table 3. Personal history of tobacco consumption was, by far, the most frequent cardiovascular risk factor in this young population presenting with premature CAD (86%).

Among the population with premature STEMI, haplogroups G and I were more common in patients with a family history of premature cardiovascular disease than in those without it (Table 4). Moreover, the haplogroup “other” was significantly more frequently found in those STEMI patients who suffered diabetes mellitus when compared to those without diabetes (Table 4).

We did not find significant differences between the haplogroups for the mean cholesterol levels (Table 5).

## 4. Discussion

Premature CAD is a significant health concern that requires early diagnosis, effective management, and ongoing prevention. It is crucial to identify and manage risk factors to prevent the development of premature CAD and its associated complications. Therefore, investigating the genetics of populations with premature CAD, presenting with life threatening events like this cohort, is of utmost importance. The heritability of CAD has been estimated to be between 40% and 60%, on the basis of family and twin studies [18]. Remarkably, these heritable effects manifest more evidently in younger individuals [19]. An interesting study performed in twins reported that the probability of dying from CAD given that one’s twin has already died from CAD decreased with increasing age, particularly amongst males [19].

Research studies have investigated the association between Y chromosome polymorphisms and the risk of CAD. However, the findings are not consistent and the relationship between Y haplogroups and CAD risk remains unclear. Charchar et al. studied three cohorts of British men with more than 3000 participants and found that carriers of haplogroup I had about a 50% higher age-adjusted risk of CAD than men with other Y haplogroups (*p* < 0.001; OR = 1.56, 1.24–1.97). The association between haplogroup I and increased risk of CAD was independent of traditional cardiovascular and socioeconomic risk factors [10]. These authors showed that this haplogroup was associated with the differential expression of genes related to inflammation and immunity, some of them relevant to atherosclerosis [10]. Haplogroup I has been also associated with a higher expression of chromosome Y genes linked to the immune system [11]. However, other authors failed to confirm the association between haplogroup I and CAD [20].

An analysis of men from the UK Biobank revealed that haplogroup I (Y1 subclade) was associated with an 11% increase in risk of CAD when compared with all other haplogroups combined (OR= 1.11, 95% CI = 1.04–1.189) [21]. The same authors showed that haplogroup-I1-specific variants showed enrichment for promoter and enhancer chromatin states in cells/tissues relevant to atherosclerosis, and haplogroup I1 was associated with changes in pathways involved in atherosclerosis development such as defense against pathogens, immunity, oxidative phosphorylation, mitochondrial respiration, lipids, coagulation, and extracellular matrix remodeling [21]. Due to the pivotal role of immunity and inflammation pathways in the development of atherosclerotic lesions, it is plausible that a differential immune response between the Y haplogroups could explain the reported association with CAD. These haplogroups might be associated with differences within pathways underlying almost all stages of CAD, including the initiation, growth, and rupture of atherosclerotic plaque [21,22,23]. A critical driver of atherosclerosis, the apolipoprotein B gene [24], was upregulated in arteries from carriers of haplogroup I1 [21]. On the contrary, CAD patients with haplogroup I1 showed the upregulation of pathways involved in platelet aggregation and arterial thrombus formation [21,25].

However, the distribution of Y chromosome haplogroups may vary from Northern to Southern European populations [8]. On the one hand, in our study, the frequency of Y haplogroup I was only slightly higher in the patients and displayed no significant difference from the controls (6% vs. 4%, *p* = 0.47, Table 2). However, it should be noted that in our population, haplogroup I was less common than in other regions where the association with CAD was reported. Moreover, the I1 subgroup is common among UK men but very rare among Spanish men (<1%). It is thus possible that the different results between populations reflect the presence of DNA variants beyond the ancestral SNP that defines the haplogroup. Thus, the sequencing of the Y-specific region is necessary to uncover the nucleotide changes that would explain the association with CAD.

On the other hand, in Southern European populations, haplogroup R1b is the most frequent (40–70%), followed by E1b1b and J2 (5–20%, respectively). In the Iberian Peninsula, the general reported prevalences are similar: 70–80% for R1B and 5–15% for E1b1b and J2, respectively [26]. In this regard, our region (Asturias) geographically belongs to the Spanish Cantabrian cornice, where haplogroup R1B is known to be the most common haplogroup. Accordingly, the frequencies from our controls were in agreement with those reported by others [26]. However, in addition, in the population of patients for whom we evaluated premature STEMI, we found significantly more patients with this particular haplogroup R1B that in the control population (Table 2).

The underlying mechanisms by which Y chromosome haplogroups influence the risk of CAD are not fully understood. However, studies have suggested that genetic variations within the Y chromosome could affect the expression of genes involved in lipid metabolism, inflammation, and thrombosis, which are key factors in the development of CAD. In this regard, Eales et al. showed that haplogroup I1 increased cardiovascular risk through the proatherosclerotic reprogramming of the transcriptome in several tissues of key relevance to CAD [21]. They observed changes in gene expression within other pathways underlying almost all stages of CAD, including the initiation, growth, and rupture of atherosclerotic plaque [21,22,23]. A critical driver of atherosclerosis, the apolipoprotein B gene [24], was upregulated in the arteries in carriers of haplogroup I1 [21]. On the contrary, CAD patients with haplogroup I1 showed the upregulation of pathways involved in platelet aggregation and arterial thrombus formation [21,25].

Moreover, while genetic factors play a significant role in the development of CAD, lifestyle factors such as smoking, poor diet, and lack of exercise also contribute to the risk of CAD. In this regard, some studies have shown that the association between Y chromosome haplogroups and CAD risk may vary depending on lifestyle factors. In this sense, a study by Pilbrow et al. found that men with the haplogroup I were at a higher risk of developing CAD if they smoked. In this regard, in this study of patients with premature CAD, the “rare” haplogroup was significantly more frequent among diabetic patients than non-diabetic ones. On the other hand, we found that haplogroup G was significantly more frequent in those patients with a family history of premature cardiovascular disease than in those without it.

## 5. Limitations

Overall, the relationship between Y chromosome haplogroups and the risk of premature CAD is complex and requires further investigation. Further studies are needed to elucidate the mechanisms underlying this association and to explore the potential clinical implications of Y chromosome haplogroups in the prevention and management of CAD. Studies with other epidemiological approaches, such as Mendelian randomization, are of interest to uncover the effect of cardio-metabolic variables in the risk for early-onset CAD. However, for this approach, a larger number of population controls and patients are required [27].

## 6. Conclusions

In conclusion, in our cohort of Spanish men, chromosome Y haplogroup R was significantly more frequent in premature STEMI. Y chromosome haplogroups may represent a novel avenue for understanding the genetic determinants of CAD risk in men.

## Figures and Tables

**Table 1:** The 8 single nucleotide polymorphisms determined to define the Y haplogroups, genotyped with Taqman assays (Fisher scientific, Hampton, NH, USA).

NUCLEOTIDE Y Position	SNP	HAPLOGROUP	ASSAY	EUR	FREQUENCY
13407103	rs2032658 G/A	R	C___2307221_1Y	G (0.58)	0.0016
12914512	rs2032624 C/A	R1	C___2292796_20	C (0.58)	0.0016
20577481	rs9786153 T/C	R1B	C__29812961_10	C (0.53)	<0.0001
2789135	rs2534636 C/T	R1A	C__26236081_10	T (0.05)	0.0113
14436668	rs113623003 G/A	I	C_153784812_10	A (0.14)	0.0085
20587967	rs13447352 A/C	J	C__33589462_10	C (0.11)	0.1168
19617112	rs9306841 C/G	E	C__29796914_10	G (0.03)	0.0008
12915617	rs2032636 G/T	G	C___2292797_20	T (0.06)	0.0996

**Table 2:** Y chromosome haplogroups differences between the male control cohort and male patients with premature coronary artery disease.

Haplogroup	Control Cohort N = 200	Patients Cohort N = 122	*p*
R	**122 (0.61)**	**88 (0.72)**	0.04 *
R1	**118 (0.59)**	**88 (0.72)**	0.02 *
R1B	**118 (0.59)**	**88 (0.72)**	0.02 *
R1A	**4 (0.02)**	**0**	0.12
I	**8 (0.04)**	**7 (0.06)**	0.47
J	**20 (0.10)**	**8 (0.07)**	0.28
E	**28 (0.14)**	**10 (0.08)**	0.12
G	**16 (0.08)**	**3 (0.02)**	0.04 *
Other	**6 (0.03)**	**6 (0.05)**	0.38

* *p* < 0.05.

**Table 3:** Clinical characteristics of patients presenting with premature ST-elevation myocardial infarction.

	122 Male Patients with Premature STEMI
Mean age at STEMI	47.11 (±8.99)
Cardiovascular risk factors	
Previous/current smoker	95 (86.07%)
High blood pressure	34 (27.87%)
Diabetes mellitus	14 (11.48%)
Dyslipidemia	45 (36.89%)
Family history of premature CAD	30 (24.59%)

STEMI: ST-elevation myocardial infarction; CAD: coronary artery disease.

**Table 4:** Y chromosome haplogroup prevalence in patients with premature cardiovascular disease, depending on the presence/absence of cardiovascular risk factors.

Y Chromosome Haplogroups	No DM (108)	DM (14)	No HTN (88)	HTN (34)	No DL (77)	DL (45)	Non-Smokers (26)	Smokers (96)	No FH of PCAD (92)	FH of PCAD (30)
R	73.2%	64.3%	70.5%	76.5%	74%	68.9%	76.9%	70.8%	71.7%	73.3%
E	9.3%	-	10.2%	2.9%	7.8%	8.9%	11.5%	7.3%	9.8%	3.3%
J	5.6%	14.3%	5.7%	8.8%	6.5%	6.7%	3.9%	7.3%	6.5%	6,7%
G	2.8%	-	3.4%	-	3.9%	-	3.9%	2.1%	1.1%	6.7% *
I	6.5%	-	6.8%	2.9%	5.2%	6.7%	3.9%	6.3%	6.5% *	3.3%
Other	2.8%	21.4% *	3.4%	8.8%	2.6%	8.9%		6.3%	4.4%	6.7%

DM: diabetes mellitus; HTN: high blood pressure; DL: dyslipidemia; FH: family history; PCAD: premature coronary artery disease. * *p* < 0.05.

**Table 5:** Mean cholesterol value levels in each Y chromosome haplogroup.

Mean Values (mg/dL)	R (*n* = 88)	E (*n* = 10)	J (*n* = 8)	G (*n* = 3)	I (*n* = 7)	Other (*n* = 6)
HDL cholesterol	37.6	42.7	39	34	39.57	33.33
LDL cholesterol	114.97	120.7	111.63	110	134.7	112.17
Triglycerides	173.30	157.2	152,88	140.67	191.86	242.5

## References

[1] Arnett D.K., Blumenthal R.S., Albert M.A., Buroker A.B., Goldberger Z.D., Hahn E.J., Himmelfarb C.D., Khera A., Lloyd-Jones D., McEvoy J.W. (2019). 2019 ACC/AHA Guideline on the Primary Prevention of Cardiovascular Disease: Executive Summary: A Report of the American College of Cardiology/American Heart Association Task Force on Clinical Practice Guidelines. J. Am. Coll. Cardiol..

[2] Visseren F.L.J., Mach F., Smulders Y.M., Carballo D., Koskinas K.C., Bäck M., Benetos A., Biffi A., Boavida J.-M., Capodanno D. (2021). 2021 ESC Guidelines on cardiovascular disease prevention in clinical practice. Eur. Heart J..

[3] Yusuf S., Hawken S., Ounpuu S., Dans T., Avezum A., Lanas F., McQueen M., Budaj A., Pais P., Varigos J. (2004). Effect of potentially modifiable risk factors associated with myocardial infarction in 52 countries (the INTERHEART study): Case-control study. Lancet.

[4] McPherson R., Tybjaerg-Hansen A. (2016). Genetics of Coronary Artery Disease. Circ. Res..

[5] Lorca R., Aparicio A., Cuesta-Llavona E., Pascual I., Junco A., Hevia S., Villazón F., Hernandez-Vaquero D., Rodríguez Reguero J.J., Moris C. (2020). Familial Hypercholesterolemia in Premature Acute Coronary Syndrome. Insights from CholeSTEMI Registry. J. Clin. Med..

[6] Jobling M.A., Tyler-Smith C. (2003). The human Y chromosome: An evolutionary marker comes of age. Nat. Rev. Genet..

[7] Underhill P.A., Shen P., Lin A.A., Jin L., Passarino G., Yang W.H., Kauffman E., Bonné-Tamir B., Bertranpetit J., Francalacci P. (2000). Y chromosome sequence variation and the history of human populations. Nat. Genet..

[8] Underhill P.A., Myres N.M., Rootsi S., Metspalu M., Zhivotovsky L.A., King R.J., Lin A.A., Chow C.-E.T., Semino O., Battaglia V. (2010). Separating the post-Glacial coancestry of European and Asian Y chromosomes within haplogroup R1a. Eur. J. Hum. Genet..

[9] Wang Z., Parikh H., Jia J., Myers T., Yeager M., Jacobs K.B., Hutchinson A., Burdett L., Ghosh A., Thun M.J. (2012). Y chromosome haplogroups and prostate cancer in populations of European and Ashkenazi Jewish ancestry. Hum. Genet..

[10] Charchar F.J., Bloomer L.D., Barnes T.A., Cowley M.J., Nelson C.P., Wang Y., Denniff M., Debiec R., Christofidou P., Nankervis S. (2012). Inheritance of coronary artery disease in men: An analysis of the role of the Y chromosome. Lancet.

[11] Bloomer L.D.S., Nelson C.P., Denniff M., Christofidou P., Debiec R., Thompson J., Zukowska-Szczechowska E., Samani N.J., Charchar F.J., Tomaszewski M. (2014). Coronary artery disease predisposing haplogroup I of the Y chromosome, aggression and sex steroids—Genetic association analysis. Atherosclerosis.

[12] Lorca R., Aparicio A., Gómez J., Álvarez-Velasco R., Pascual I., Avanzas P., González-Urbistondo F., Alen A., Vázquez-Coto D., González-Fernández M. (2023). Mitochondrial Heteroplasmy as a Marker for Premature Coronary Artery Disease: Analysis of the Poly-C Tract of the Control Region Sequence. JCM.

[13] Thygesen K., Alpert J.S., Jaffe A.S., Chaitman B.R., Bax J.J., Morrow D.A., White H.D., ESC Scientific Document Group (2019). Fourth universal definition of myocardial infarction (2018). Eur. Heart J..

[14] Collet J.-P., Thiele H., Barbato E., Barthélémy O., Bauersachs J., Bhatt D.L., Dendale P., Dorobantu M., Edvardsen T., Folliguet T. (2021). 2020 ESC Guidelines for the management of acute coronary syndromes in patients presenting without persistent ST-segment elevation. Eur. Heart J..

[15] Williams R.R., Hunt S.C., Schumacher M.C., Hegele R.A., Leppert M.F., Ludwig E.H., Hopkins P.N. (1993). Diagnosing heterozygous familial hypercholesterolemia using new practical criteria validated by molecular genetics. Am. J. Cardiol..

[16] Defesche J.C., Lansberg P.J., Umans-Eckenhausen M.A.W., Kastelein J.J.P. (2004). Advanced method for the identification of patients with inherited hypercholesterolemia. Semin. Vasc. Med..

[17] Riobello C., Gómez J., Gil-Peña H., Tranche S., Reguero J.R., de la Hera J.M., Delgado E., Calvo D., Morís C., Santos F. (2016). KCNQ1 gene variants in the risk for type 2 diabetes and impaired renal function in the Spanish Renastur cohort. Mol. Cell. Endocrinol..

[18] Vinkhuyzen A.A.E., Wray N.R., Yang J., Goddard M.E., Visscher P.M. (2013). Estimation and Partition of Heritability in Human Populations Using Whole-Genome Analysis Methods. Annu. Rev. Genet..

[19] Zdravkovic S., Wienke A., Pedersen N.L., Marenberg M.E., Yashin A.I., De Faire U. (2002). Heritability of death from coronary heart disease: A 36-year follow-up of 20,966 Swedish twins. J. Intern. Med..

[20] Timmers P.R.H.J., Wilson J.F. (2022). Limited Effect of Y Chromosome Variation on Coronary Artery Disease and Mortality in UK Biobank-Brief Report. Arterioscler. Thromb. Vasc. Biol..

[21] Eales J.M., Maan A.A., Xu X., Michoel T., Hallast P., Batini C., Zadik D., Prestes P.R., Molina E., Denniff M. (2019). Human Y Chromosome Exerts Pleiotropic Effects on Susceptibility to Atherosclerosis. Arterioscler. Thromb. Vasc. Biol..

[22] Newby A.C. (2015). Metalloproteinases promote plaque rupture and myocardial infarction: A persuasive concept waiting for clinical translation. Matrix Biol..

[23] Ghattas A., Griffiths H.R., Devitt A., Lip G.Y.H., Shantsila E. (2013). Monocytes in Coronary Artery Disease and Atherosclerosis. J. Am. Coll. Cardiol..

[24] Borén J., Williams K.J. (2016). The central role of arterial retention of cholesterol-rich apolipoprotein-B-containing lipoproteins in the pathogenesis of atherosclerosis: A triumph of simplicity. Curr. Opin. Lipidol..

[25] Evans D.J.W., Jackman L.E., Chamberlain J., Crosdale D.J., Judge H.M., Jetha K., Norman K.E., Francis S.E., Storey R.F. (2009). Platelet P2Y_12_ Receptor Influences the Vessel Wall Response to Arterial Injury and Thrombosis. Circulation.

[26] Adams S.M., Bosch E., Balaresque P.L., Ballereau S.J., Lee A.C., Arroyo E., López-Parra A.M., Aler M., Grifo M.S.G., Brion M. (2008). The genetic legacy of religious diversity and intolerance: Paternal lineages of Christians, Jews, and Muslims in the Iberian Peninsula. Am. J. Hum. Genet..

[27] Bell K.J.L., Loy C., Cust A.E., Teixeira-Pinto A. (2021). Mendelian Randomization in Cardiovascular Research: Establishing Causality When There Are Unmeasured Confounders. Circ. Cardiovasc. Qual. Outcomes.

